# Preventive Intrathecal Injection of Bupivacaine Alleviated Microglia Activation and Neuropathic Pain in a Rat Model of Chronic Constriction Injury

**DOI:** 10.3390/ijms23137197

**Published:** 2022-06-28

**Authors:** Chih-Cheng Wu, Cheng-Yi Chang, Chung-Yuh Tzeng, Jen-Hsuan Huang, Chih-Jen Hung, Wen-Ying Chen, Su-Lan Liao, Yu-Hsiang Kuan, Chun-Jung Chen

**Affiliations:** 1 Department of Anesthesiology, Taichung Veterans General Hospital, Taichung City 407, Taiwan; chihcheng.wu@gmail.com (C.-C.W.); hung.chihjen@gmail.com (C.-J.H.); 2 Department of Financial Engineering, Providence University, Taichung City 433, Taiwan; 3 Department of Data Science and Big Data Analytics, Providence University, Taichung City 433, Taiwan; 4 Department of Surgery, Feng Yuan Hospital, Taichung City 420, Taiwan; c.y.chang.ns@gmail.com; 5 Department of Veterinary Medicine, National Chung-Hsing University, Taichung City 402, Taiwan; wychen@dragon.nchu.edu.tw; 6 Department of Orthopedics, Taichung Veterans General Hospital, Taichung City 407, Taiwan; tcy@vghtc.gov.tw; 7 Department of Medicinal Botanicals and Health Applications, Da-Yeh University, Changhua County 515, Taiwan; 8 Department of Anesthesiology, Show Chwan Memorial Hospital, Changhua County 500, Taiwan; noel-jh@yahoo.com.tw; 9 Department of Medical Research, Taichung Veterans General Hospital, Taichung City 407, Taiwan; slliao@vghtc.gov.tw; 10 Department of Pharmacology, Chung Shan Medical University, Taichung City 402, Taiwan; kuanyh@csmu.edu.tw; 11 Department of Medical Laboratory Science and Biotechnology, China Medical University, Taichung City 404, Taiwan

**Keywords:** anesthetics, microglia, neuropathic pain, sciatic nerve

## Abstract

Spinal microglia are crucial to neuronal hyper-excitability and pain hypersensitivity. The local anesthetic bupivacaine is commonly used for both peripheral and spinal anesthesia. The pain-relief effects resulting from the peripheral and systemic administration of bupivacaine have been previously reported. In this study, the preventive effects of intrathecal bupivacaine administration against neuropathic pain were revealed in a rat model of sciatic nerve chronic constriction injury (CCI). Using a CCI rat model, pain hypersensitivity, characterized by mechanical allodynia and thermal hyperalgesia, correlated well with microglia M1 polarization, activation and pro-inflammatory cytokine expression in both spinal cord dorsal horns and sciatic nerves. Bupivacaine attenuated pain behaviors and inflammatory alternations. We further identified that the Interferon Regulatory Factor 5 (IRF5)/P2X Purinoceptor 4 (P2X4R) and High Mobility Group Box 1 (HMGB1)/Toll-Like Receptor 4 (TLR4)/NF-κB inflammatory axes may each play pivotal roles in the acquisition of microglia M1 polarization and pro-inflammatory cytokine expression under CCI insult. The relief of pain paralleled with the suppression of microglia M1 polarization, elevation of microglia M2 polarization, and inhibition of IRF5/P2X4R and HMGB1/TLR4/NF-κB in both the spinal cord dorsal horns and sciatic nerve. Our findings provide molecular and biochemical evidence for the anti-neuropathic effect of preventive bupivacaine.

## 1. Introduction

Clinical treatment of neuropathic pain remains a challenging obstacle for those in the medical field. Lesions, dysfunction, and diseases centered on the central or peripheral nerve system are all common causes of persistent and chronic neuropathic pain [1]. Those intolerable and painful conditions caused by neuropathic pain greatly reduce the quality of life for the victim patients and predispose them to suffer from mood disorders [2]. Although NSAIDs, tricyclic antidepressants, opioids, and gabapentin have all been prescribed and recommended to patients experiencing neuropathic pain, clinical management of the condition remains unsatisfactory [3]. To overcome such obstacles and uncover possible therapeutic options, the elucidation of pathogenic mechanisms and identification of novel or supplementary strategies are needed.

Lesions or diseases affecting the somatosensory nerve system, particularly the neurons, are the main mechanisms underlying the development and maintenance of neuropathic pain [1]. Alternatively, studies have revealed that spinal microglia are key cells involved in neuropathic pain and further highlight the substantial and deteriorative roles of neuroinflammation. In the spinal cord, microglia respond to local insults, retrograde transported bioactive molecules, or peripheral nerve injury by shifting to an M1 polarization pro-inflammatory phenotype and the releasing of cytokines, chemokines, and algesic substances. Activated microglia and their accompanied pro-inflammatory mediators orchestrate algesic conditions by favoring central and peripheral sensitization, neuronal hyper-excitability, and pain transmission, leading to allodynia and hyperalgesia [4,5,6,7,8,9]. Therefore, microglia and neuroinflammation have become emerging targets for the therapeutic relief of neuropathic pain.

Local anesthetics are commonly used for the relief of nociceptive pain [10]. Clinically, bupivacaine is the most commonly used local anesthetic for spinal anesthesia. The peripheral and systemic administration of bupivacaine has been shown to alleviate pain behaviors in rodent models of thoracotomy, spared nerve injury (SNI), spinal nerve ligation (SNL), and complete Freund’s adjuvant foot injection [11,12,13,14]. Additionally, the use of bupivacaine for nerve blocking decreases the axonal transport of pro-inflammatory mediators in rat models of carrageenan-induced inflammation [15]. Additionally, the intrathecal injection of bupivacaine over a course of 3 days improves painful diabetic neuropathy with concurrent microglia inhibition [16]. Despite the advances seen in pain studies, the effects and beneficial mechanisms of bupivacaine preventive treatment against neuropathic pain remain largely unclear.

Rodent models of peripheral nerve injury such as SNI, SNL, and chronic constriction injury (CCI) have been well established for the study of neuropathic pain. SNI produces long-lasting neuropathic pain-like behaviors by cutting the tibial and common peroneal nerve branches of the sciatic nerve. SNL is established by tightly ligating the 5th and 6th lumbar nerves (L5/L6) with silk thread [11,12]. Among the models, sciatic nerve unilateral CCI, a low-trauma surgical procedure, simulates spontaneous pain, allodynia, and hyperalgesia with concurrent spinal and sciatic nerve inflammation [4,5,7,17]. In this study, the effects of bupivacaine preventive intrathecal injection on neuropathic pain in a rat model of sciatic nerve CCI were investigated, focusing on microglia polarization and activation.

## 2. Results

### 2.1. Bupivacaine Alleviated Pain Behaviors

**Figure 1:**
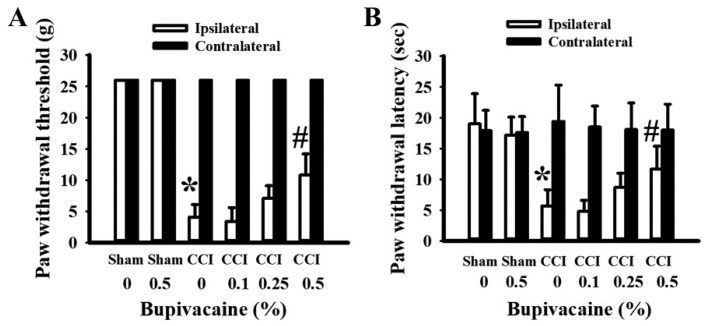
Bupivacaine alleviated mechanical allodynia and thermal hyperalgesia in CCI rats. Rats were intrathecally injected with the indicated concentrations of bupivacaine (0–0.5%, diluted in saline) for 30 min prior to being subjected to sham and CCI operation. Seven days later, mechanical allodynia (**A**) and thermal hyperalgesia (**B**) were evaluated in the ipsilateral and contralateral limbs. * *p* < 0.05 vs. sham/saline/ipsilateral group and # *p* < 0.05 vs. CCI/saline/ipsilateral group, *n* = 6.

To evaluate the preventive potential of bupivacaine against neuropathic pain, various concentrations of bupivacaine (0–0.5%) were intrathecally delivered. Unlike the rats used in the sham operations, CCI rats tended to show a decrease in the paw withdrawal threshold to a filament stimulus (Figure 1A), as well as lifting and suspending their right hind paws away from the heat plate (Figure 1B). In contrast, the paw mechanical withdrawal threshold (Figure 1A) and thermal withdrawal latency (Figure 1B) of the contralateral hind paws did not change among the groups. Although bupivacaine at a concentration of 0.5% displayed limited effects on the sham rats, it prolonged the paw mechanical withdrawal threshold (Figure 1A) and thermal withdrawal latency (Figure 1B) in CCI rats. These findings indicate that preventive bupivacaine intrathecal treatment alleviated pain behaviors in CCI rats.

### 2.2. Bupivacaine Alleviated Microglia Activation

**Figure 2:**
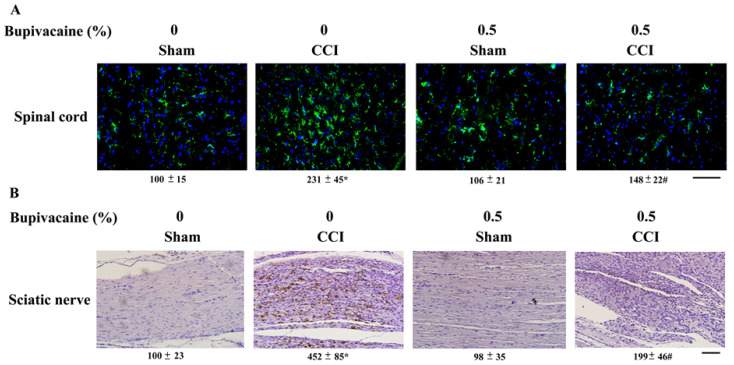
Bupivacaine alleviated microglia immunoreactivity in CCI rats. Rats were intrathecally injected with the indicated concentrations of bupivacaine (0 and 0.5%, diluted in saline) for 30 min prior to being subjected to sham and CCI operation. Seven days later, frozen sections of ipsilateral spinal cords (L4–L6) (**A**) and paraffin sections of ipsilateral sciatic nerves (**B**) were prepared and subjected to immunohistochemistry with antibodies recognizing CD68. Scale bar: 50 μm. Representative photomicrographs show immunoreactivity visualized with FITC fluorescence ((**A**), counterstained with Hoechst 33342) and diaminobenzidine ((**B**), counterstained with hematoxylin), respectively. Relative intensity of immunoreactivity was measured and depicted under photomicrographs (%). * *p* < 0.05 vs. sham/saline group and # *p* < 0.05 vs. CCI/saline group, *n* = 3.

To reveal signs of microglia activation, immunohistochemical examination of macrophages/microglia-related cluster of differentiation 68 (CD68) immunoreactivity, a representative marker of macrophages/microglia activation, was first performed. Based on the measurements of immunoreactivity intensity, there was detectable basal CD68 immunoreactivity in the ipsilateral spinal cord dorsal horns of sham rats receiving either saline (100 ± 15%) or bupivacaine (106 ± 21%). An increase in CD68 immunoreactivity was detected in the ipsilateral spinal cord dorsal horns of CCI rats (231 ± 45%), and the increment was alleviated by bupivacaine (148 ± 22%) (Figure 2A). Similar changes in CD68 immunoreactivity were found in the ipsilateral sciatic nerves of CCI rats after bupivacaine treatment, from 452 ± 85% to 199 ± 46% (Figure 2B). Data taken from an immunohistochemical examination revealed both spinal and sciatic microglia activation in CCI rats and an alleviative effect due to bupivacaine.

### 2.3. Bupivacaine Alleviated Inflammation in the Spinal Cords

**Figure 3:**
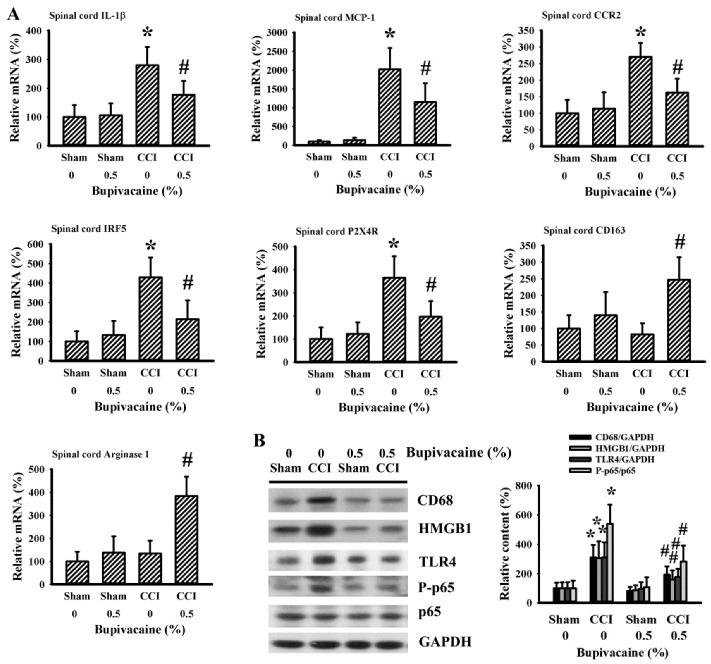
Bupivacaine alleviated inflammatory responses in the spinal cords of CCI rats. Rats were intrathecally injected with the indicated concentrations of bupivacaine (0 and 0.5%, diluted in saline) for 30 min prior to being subjected to sham and CCI operation. Seven days later, ipsilateral spinal cord dorsal horns (L4–L6) were collected and subjected to quantitative RT-PCR for the measurement of IL-1β, MCP-1, CCR2, IRF5, P2X4R, CD163, and arginase 1 mRNA expression (**A**). Protein content of CD68, HMGB1, TLR4, P-p65, p65, and GAPDH was measured in the ipsilateral spinal cord dorsal horns (L4–L6) using Western blotting. Representative blots and quantitative results are shown (**B**). * *p* < 0.05 vs. sham/saline group and # *p* < 0.05 vs. CCI/saline group, *n* = 6.

To correlate the immunohistochemical findings of microglia activation in the ipsilateral spinal cord dorsal horns of CCI rats, the parameters of microglia polarization and activation were further examined. In assessing spinal cord dorsal horn tissues, elevated expressions of inflammatory cytokine Interleukin-1β (IL-1β), immune cell recruitment-related Monocyte Chemoattractant Protein-1 (MCP-1) and CC Chemokine Receptor 2 (CCR2), microglia M1 polarization-related Interferon Regulatory Factor 5 (IRF5) and P2X Purinoceptor 4 (P2X4R) mRNA were detected in the ipsilateral side 7 days after CCI, with the changes being alleviated in CCI rats upon bupivacaine treatment (Figure 3A). Parallel changes were found in the protein levels of macrophages/microglia-related CD68, inflammation-related High Mobility Group Box 1 (HMGB1), Toll-Like Receptor 4 (TLR4), and P-p65 (Figure 3B). Although the levels of microglia M2 polarization-related CD163 and arginase 1 mRNA had not changed between the sham and CCI rats, their expression increased in CCI rats that had undergone bupivacaine treatment (Figure 3A). Biochemical findings reveal suppression of microglia M1 polarization, elevation of microglia M2 polarization, and inhibition of the HMGB1/TLR4/NF-κB inflammatory axis in the spinal cord dorsal horns of CCI rats that had undergone preventive treatment with bupivacaine.

### 2.4. Bupivacaine Alleviated Inflammation in the Sciatic Nerve

**Figure 4:**
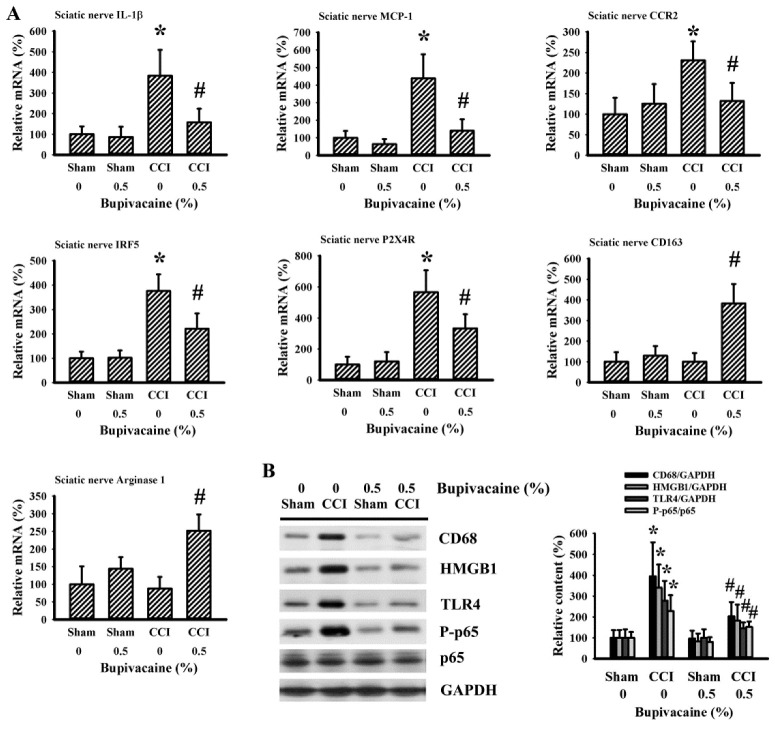
Bupivacaine alleviated inflammatory responses in the sciatic nerves of CCI rats. Rats were intrathecally injected with the indicated concentrations of bupivacaine (0 and 0.5%, diluted in saline) for 30 min prior to being subjected to sham and CCI operation. Seven days later, ipsilateral sciatic nerve tissues were collected and subjected to quantitative RT-PCR for the measurement of IL-1β, MCP-1, CCR2, IRF5, P2X4R, CD163, and arginase 1 mRNA expression (**A**). Protein content of CD68, HMGB1, TLR4, P-p65, p65, and GAPDH was measured in the ipsilateral sciatic nerve tissues using Western blotting. Representative blots and quantitative results are shown (**B**). * *p* < 0.05 vs. sham/saline group and # *p* < 0.05 vs. CCI/saline group, *n* = 6.

Because the sciatic nerve was the site of trauma in CCI rats, the alternations in microglia polarization and activation in the ipsilateral sciatic nerve tissues were examined as well. Parallel alternations were detected in the mRNA levels of IL-1β, MCP-1, CCR2, IRF5, P2X4R, CD163, and arginase 1 (Figure 4A), as well as the protein levels of CD68, HMGB1, TLR4, and P-p65 (Figure 4B). Therefore, bupivacaine still displayed suppression of microglia M1 polarization, elevation of microglia M2 polarization, and inhibition of the HMGB1/TLR4/NF-κB inflammatory axis in the sciatic nerve of CCI rats.

## 3. Discussion

Current treatments for persistent and chronic neuropathic pain are not yet satisfactory, and the lack of early prevention approaches may be one possible reason as to why. Bupivacaine is a local anesthetic for most locoregional procedures, with 0.5% isobaric bupivacaine being frequently prescribed for peripheral and spinal anesthesia. The action of bupivacaine is attributable to sodium channel blockade, N-methyl-D-aspartate blockade, or anti-inflammation [11]. Pain-relief effects from the peripheral and systemic administration of bupivacaine have been reported [11,12,13,14,15,16]. In this study, the preventive effects of intrathecal bupivacaine administration against neuropathic pain were revealed in a rat model of sciatic nerve CCI. The relief of pain behaviors attributed to bupivacaine paralleled with the suppression of microglia M1 polarization, elevation of microglia M2 polarization, and inhibition of the HMGB1/TLR4/NF-κB inflammatory axis in the spinal cord dorsal horns and sciatic nerve. These findings further provide both molecular and biochemical evidence for the anti-neuropathic effect of preventive bupivacaine.

Hyper-excitation and the abnormal repetitive firing of a subset of sensory neurons are considered to be key components in the underlying pathophysiology associated with neuropathic pain. Accumulating evidence has indicated that spinal microglia activation and microgliosis are both crucial to such neuronal hyper-excitability and pain hypersensitivity [8]. Microglia are the resident immune cells of the central nervous system (CNS) for immune surveillance and homeostasis. Under pathophysiological conditions, microglia adopt distinct reactive phenotypes through a complicated network of intracellular and extracellular signals, transcriptional programs, and gene profiles. Microglia M1 polarization is a classically activated phenotype and contributes to inflammation by releasing pro-inflammatory mediators, with the result of immune cell recruitment and activation, as well as tissue destruction. On the contrary, anti-inflammatory microglia M2 polarization, an alternatively activated phenotype, is associated with a high expression of CD163 and arginase 1, resulting in suppressive cytokine expression, phagocytosis, and tissue repair [5,6,7]. A shift towards microglia M1 polarization and a decline of microglia M2 polarization is revealed in the spinal cord dorsal horns, correlating well with neuropathic pain progression [5,7,18]. Studies have shown the inhibitory effects bupivacaine has on microglia [12,14,16]. In CCI rats, pain behaviors such as mechanical allodynia and thermal hyperalgesia paralleled with higher spinal CD68 immunohistochemical reactivity, CD68 protein content, and IL-1β, MCP-1, and CCR2 mRNA levels. Bupivacaine alleviated these changes and caused an additional upregulation of both CD163 and arginase 1 mRNA expression in CCI rats. Elevated NLRP3 inflammasome/IL-1β and MCP-1/CCR2-guided immune cell infiltration and activation each play pivotal roles in the development and progression of neuropathic pain. Their suppression attenuates microglia activation and pain behaviors, and correlates well with pain-relief treatments [9,19,20]. Therefore, spinal microglia could be an active target of bupivacaine in relieving neuropathic pain under CCI conditions.

The activated states of microglia are tightly controlled by membrane-bound surface receptors and intracellular transcription factors. Among the families of purinergic ionotropic receptors and Interferon Regulatory Factors, both IRF5 and P2X4R have been indicated in microglia activation and the development of neuropathic pain via an IRF5/P2X4R axis [6,8]. Evidence also indicates there is crosstalk between P2X4R and TLR4 in pain hypersensitivity [21]. HMGB1, a nuclear DNA-binding protein, is an endogenous ligand of TLR4. The engagement of HMGB1 and TLR4 creates a pro-inflammatory condition by triggering the p65 NF-κB transcription axis. Such activation of the HMGB1/TLR4/NF-κB axis predisposes the acquisition of microglia M1 polarization and inflammatory cytokine expression leading to pain hypersensitivity [22]. In the spinal cord dorsal horn tissues of CCI rats, elevated mRNA levels of IRF5 and P2X4R, along with an increased protein content of HMGB1 and TLR4, as well as p65 protein phosphorylation were detected. Upon gathering relevant studies and putting them together with our findings, we determined that IRF5/P2X4R and HMGB1/TLR4/NF-κB axes working together in a concerted link or as an independent mechanism play substantial roles in guiding spinal microglia M1 polarization and pro-inflammatory cytokine expression. Their actions contributed to the development of neuropathic pain in CCI rats and represented targets of intervention for bupivacaine.

After peripheral nerve injury occurs, the sites of damage are the origins of the synthesis and export of biologically active molecules. Data taken from immunohistochemical examination and molecular analyses revealed a similar alternation in microglia polarization, pro-inflammatory cytokine mRNA expression, IRF5/P2X4R, and HMGB1/TLR4/NF-κB in the sciatic nerve tissues of CCI rats. Bupivacaine nerve block shows an inhibitory effect on the retrograde transport of pro-inflammatory mediators [15]. Therefore, the suppression of spinal microglia activation and pain hypersensitivity in CCI rats through the administration of bupivacaine may be secondary to its effects on the sciatic nerve. Currently, the direct spinal effects, secondary to the sciatic nerve effects, or both, could be the underlying pain-relieving effects of bupivacaine, although this has not yet been determined.

Although the current study has provided both molecular and biochemical evidence showing the preventive effects of bupivacaine against CCI neuropathic pain, certain parts of the supporting data were indirect. Instead of protein levels, data regarding IL-1β, MCP-1, CCR2, IRF5, P2X4R, CD163, and arginase 1 were analyzed at the mRNA level. Additionally, the characterization of microglia polarization by flow cytometry or immunohistochemical identification would provide more solid evidence. These limitations weaken the translational implications taken from the current findings.

Independent of etiologies, injuries to peripheral nerves can cause persistent and chronic neuropathic pain. Unfortunately, current treatments for persistent and chronic neuropathic pain are not satisfactory [1,2,3]. It is proposed that early prevention might provide an opportunity to achieve a better outcome. There are studies showing that systemic and intrathecal administration of bupivacaine has benefits in different types of pain behaviors [14,15,16]. In this study, we further revealed that preventive intrathecal injection of bupivacaine improved pain behaviors in a rat model of CCI. Because bupivacaine is commonly used for spinal anesthesia, our findings suggest that early prevention with bupivacaine represents a candidate for the prevention and control of neuropathic pain, particularly in post-operative pain. It should be noted that bupivacaine might induce neurotoxic effects due to intraneural local anesthetic injection. In addition, overdoses of bupivacaine are also toxic to the CNS and cardiovascular system [23]. Therefore, clinically relevant administrative protocols and regimens centered on bupivacaine should be further investigated.

Bupivacaine is commonly used for both peripheral and spinal anesthesia. For spinal anesthesia, three milliliters of bupivacaine at a concentration of 0.5% for an adult (50 kg body weight) is recommended. Bupivacaine at concentrations of 0.1% and 0.25% is prescribed to an adult for peripheral anesthesia, whereas volumes depend on the types of nerves and severity of pain. Using sciatic nerve CCI rat models, pain hypersensitivity correlated well with microglia M1 polarization, activation, and pro-inflammatory cytokine expression in both spinal cord dorsal horns and sciatic nerves. Fifteen microliters of intrathecal bupivacaine (0.5%) preventive administration attenuated pain behaviors and inflammatory alternations in CCI rats (250 g body weight). Microglia activation and neuroinflammation are cues surrounding neuronal hyper-excitability and pain hypersensitivity. We further identified that the IRF5/P2X4R and HMGB1/TLR4/NF-κB inflammatory axes may be pivotal to the acquisition of microglia M1 polarization and pro-inflammatory cytokine expression under CCI insult. Although limitations still remain and further investigation is warranted, those pro-inflammatory alternations centered on microglia could be direct or indirect targets of bupivacaine in the search for pain relief.

## 4. Materials and Methods

### 4.1. Animal Allocation and Treatments

**Figure 5:**
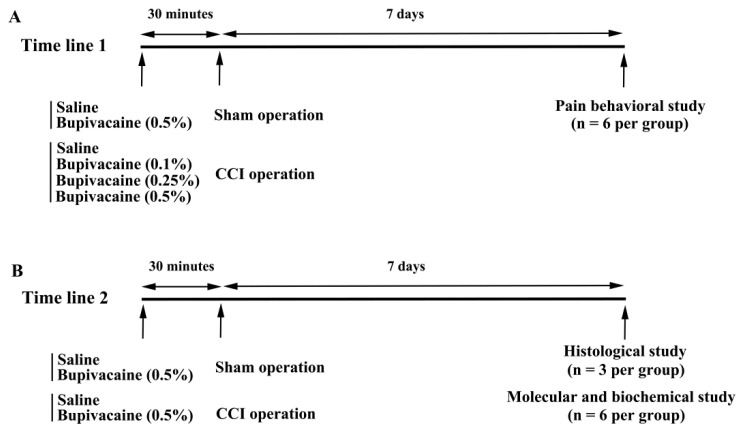
Time lines for experimental procedures. (**A**) Pain behavioral study consisted of six groups, including two groups of sham operation and four groups of CCI operation, indicated as time line 1. (**B**) Time line 2 was drawn for histological study and molecular/biochemical study consisting of two groups of sham operation and two groups of CCI operation.

The sciatic nerve CCI model was produced in adult male Sprague-Dawley rats (age 8 weeks) according to reported procedures [18,19,20]. All experimental procedures were performed following the approval of The Animal Experimental Committee of Taichung Veterans General Hospital (IACUC approval code: La-1051415, IACUC approval date: 23 November 2016). Rats (BioLASCO Taiwan Co., Ltd., Taipei, Taiwan) were housed in a standard conditioned animal facility with free access to rat chow and tap water. Under isoflurane anesthesia (2–4%), muscles on the right inguinal region were gently separated and the sciatic nerve exposed at mid-thigh level without stretching muscles and nerves. Proximal to the sciatic trifurcation, the nerve was loosely tied with four ligatures using 4–0 chromic catgut approximately 1.0 mm apart. Slight depression of the nerve was appropriate for ligation intensity. The muscles and skin were then sutured and disinfected, and the rats returned to cages. For sham operations, all steps remained the same except for sciatic nerve ligation. Intrathecal bupivacaine administration was performed 30 min prior to both the sham and CCI operations. A needle was inclined to an angle of approximately 30° and then inserted into the intervertebral space. A successful piercing of the dura was evidenced by a reflexive swing of the tail. Saline and bupivacaine were delivered through a 30-gauge sterile needle inserted vertically into the L5–L6 vertebrae at a volume of 15 microliters, followed by an additional 15 microliters of saline, as performed in a previously reported method [18]. For pain behavior study, rats were allocated into six groups (*n* = 6 per group): sham group receiving saline, sham group receiving bupivacaine (5 mg/mL, 0.5%), CCI group receiving saline, and 3 CCI groups receiving bupivacaine (1 mg/mL (0.1%), 2.5 mg/mL (0.25%), and 5 mg/mL (0.5%)). For molecular and biochemical study, rats were divided into four groups (*n* = 6 per group): sham group receiving saline, sham group receiving bupivacaine (5 mg/mL, 0.5%), CCI group receiving saline, and CCI group receiving bupivacaine (5 mg/mL, 0.5%). Because CCI in rodents caused severe pain behaviors on day 7 after surgery [18,19,20], all animals were euthanized and subjected to further analyses 7 days after surgical procedures were performed (Figure 5).

### 4.2. Pain Behavioral Measurement

Pain behavioral measurements were performed in accordance with the reported methods, with slight modifications [19,20]. Rats were placed on a perforated grid for 5 min before a mechanical allodynia measurement was performed using a von Frey filament in ascending force (1, 2, 4, 6, 8, 10, 15, and 26 g). The paw mechanical withdrawal threshold was defined and set as at least a three-foot withdrawal in five trials at each force category. The paw thermal withdrawal latency was determined using a heat plate at a temperature of 54 ± 0.5 °C. The time when the hind paws lifted from, and became suspended over, the plate was recorded.

### 4.3. Immunohistochemical Examination

Immunohistochemical examination was performed in accordance with the reported methods, with slight modifications [24]. Frozen sections (8 μm) of dissected spinal cords (L4–L6) were incubated with antibodies against CD68 (1:100, Serotec, Raleigh, NC, USA), and the cell nuclei stained with Hoechst 33342. The immunoreactive signals were visualized using FITC-conjugated IgG under an epifluorescence microscope. Paraffin-embedded sciatic nerve specimens were prepared for subsequent immunohistochemical examination. Immunohistochemistry of the deparaffinized sections (8 μm) was performed by incubation with the CD68 antibodies, and then visualized using diaminobenzidine as the chromogenic substrate. The intensity of immunoreactivity was analyzed using Image J software (NIH, Bethesda, MD, USA).

### 4.4. RNA Isolation and Quantitative Real-Time Reverse Transcriptase Polymerase Chain Reaction (RT-PCR)

The procedures for tissue RNA extraction, complementary DNA synthesis, quantitative real-time PCR and calculation were performed as previously described [25]. Tissues from both the right sciatic nerve (10 mm in length) and right spinal cord dorsal horn (L4–L6) were collected for the measurement of mRNA levels. The primers used in the PCR amplification were: 5′-CACCTCTCAAGCAGAGCACAG and 5′-GGGTTCCATGGTGAAGTCAAC for IL-1β (NM_031512.2); 5′-GTTGTTCACAGTTGCTGCCT and 5′-CTCTGTCATACTGGTCACTTCTAC for MCP-1 (NM_031530.1); 5′-AGAGAGCTGCAGCAAAAAGG and 5′-GCAAAGAGGCAGTTGCAAAG for CCR2 (NC_051345.1); 5′-GGAGTAGGGAGGATGTTTATTGG and 5′-AACTACTACCAAACCACCRCTCC for IRF5 (NC_051351.1); 5′-GGGTGAAGTTTTATTCCAGC and 5′-GGGTGAAGTTTTCTGCAGCC for P2X4R (NC_051350.1); 5′-CCAGTCCCAAACACTGTCCT and 5′-ATGCCAGTGAGCTTCCCGTTCAGC for (CD163, NC_051342.1); 5′-GGAATCTGCATGGGCAACCTGTGT and 5′-AGGGTCTACGTCTCGCAAGCCA for (arginase 1, NC_051344.1); 5′-AAGTCCCTCACCCTCCCAAAAG and 5′-AAGCAATGCTGTCACCTTCCC for β-actin (NC_051345.1).

### 4.5. Western Blot

The procedures for tissue protein extraction, protein concentration determination, SDS-PAGE, antibody reaction and band intensity quantitation were performed as previously described [25]. Tissues from the right sciatic nerve (10 mm in length) and right spinal cord dorsal horn (L4–L6) were collected for the measurement of protein contents. The targets of primary antibodies recognized were: CD68 (1:1000), HMGB1 (1:1000), TLR4 (1:1000), Phospho-p65 (P-p65, 1:500), p65 (1:1000) and Glyceraldehyde-3-Phosphate Dehydrogenase (GAPDH, 1:3000) (Santa Cruz Biotechnology, Santa Cruz, CA, USA).

### 4.6. Statistical Analysis

Experimental data were analyzed using GraphPad Prism software (San Diego, CA, USA). Statistical significance was defined as *p* values less than 0.05. Data shown in this study were expressed as mean values ± standard deviation. Two-way analysis of variance (ANOVA) was performed to evaluate experimental values between groups, whereas Dunnett’s post-hoc test was used to assess comparisons.

## Data Availability

Not applicable.
